# Effect of Hydrothermal Treatment and Doping on the Microstructural Features of Sol-Gel Derived BaTiO_3_ Nanoparticles

**DOI:** 10.3390/ma14154345

**Published:** 2021-08-03

**Authors:** Nico Zamperlin, Riccardo Ceccato, Marco Fontana, Alessandro Pegoretti, Andrea Chiappini, Sandra Dirè

**Affiliations:** 1 Department of Industrial Engineering, University of Trento, Via Sommarive 9, 38123 Trento, Italy; riccardo.ceccato@unitn.it (R.C.); marco.fontana@santannapisa.it (M.F.); alessandro.pegoretti@unitn.it (A.P.); sandra.dire@unitn.it (S.D.); 2 Institute of Mechanical Intelligence, Scuola Superiore Sant’Anna, Piazza Martiri della Libertà 33, 56127 Pisa, Italy; 3 National Interuniversity Consortium of Materials Science and Technology (INSTM), Via G. Giusti, 9, 50121 Firenze, Italy; 4 Institute of Photonics and Nanotechnologies (IFN-CNR), CSMFO Laboratory and Fondazione Bruno Kessler (FBK) Photonics Unit, Via alla Cascata 56/C, Povo, 38123 Trento, Italy; chiappini@fbk.eu

**Keywords:** piezoceramics, nanoparticles, sol-gel, hydrothermal treatment, doping

## Abstract

Barium Titanate (BaTiO_3_) is one of the most promising lead-free ferroelectric materials for the development of piezoelectric nanocomposites for nanogenerators and sensors. The miniaturization of electronic devices is pushing researchers to produce nanometric-sized particles to be embedded into flexible polymeric matrices. Here, we present the sol-gel preparation of crystalline BaTiO_3_ nanoparticles (NPs) obtained by reacting barium acetate (Ba(CH_3_COO)_2_) and titanium (IV) isopropoxide (Ti(O^i^Pr)_4_). The reaction was performed both at ambient conditions and by a hydrothermal process carried on at 200 °C for times ranging from 2 to 8 h. Doped BaTiO_3_ nanoparticles were also produced by addition of Na, Ca, and Bi cations. The powders were annealed at 900 °C in order to improve NPs crystallinity and promote the cubic-to-tetragonal (c⟶t) phase transformation. The microstructural features of nanoparticles were investigated in dependence of both the hydrothermal reaction time and the presence of dopants. It is found that short hydrothermal treatment (2 h) can produce BaTiO_3_ spherical and more homogeneous nanoparticles with respect to longer hydrothermal treatments (4 h, 6 h, 8 h). These particles (2 h) are characterized by decreased dimension (approx. 120 nm), narrower size distribution and higher tetragonality (1.007) in comparison with particles prepared at ambient pressure (1.003). In addition, the short hydrothermal treatment (2 h) produces particles with tetragonality comparable to the one obtained after the longest process (8 h). Finally, dopants were found to affect to different extents both the c⟶t phase transformation and the crystallite sizes.

## 1. Introduction

During the last few years, there has been an increasing interest in the development of piezoelectric composite materials for energy harvesting, actuators, and soft sensors applications. The coupling of piezo-ceramic fillers with polymeric matrices, such as polydimethylsiloxane (PDMS) and polyvinylidenefluoride (PVDF), can combine high dielectric and piezoelectric properties of ceramics with flexibility and softness of polymers [1,2,3]. To get high performance piezo-composites, the production and development of piezoceramics plays a key role. Up to the last decade, one of the most widely used piezoelectric materials was lead-zirconium titanate (PZT) [4,5], a metallic oxide with a perovskitic structure that possesses very high dielectric and piezoelectric properties [6,7]. However, there are several issues concerning this material due to the presence of lead (Pb). Pb is harmful both for the environment and human health; moreover, additional problems are related to its correct disposal. In this scenario, in the last few years, researchers have been pushing on to find a valid, high performance, lead-free alternative to PZT [8,9]. The most widely studied Pb-free piezoelectric ceramics are titanates (in particular barium-strontium titanate (Ba_1−x_Sr_x_TiO_3_) and barium titanate (BaTiO_3_)) [10] and niobates (lithium niobate, LiNbO_3_, sodium niobate NaNbO_3_, and potassium niobate KNbO_3_) [9,11,12]. 

BaTiO_3_ (BT) is one of the most promising lead-free perovskitic oxides. It was largely employed before the discovery of PZT and it is now facing a renewed interest due to its high permittivity and ferroelectric and piezoelectric properties [13]. Barium titanate presents the typical perovskitic structure ABO_3_ (A and B are metallic cations) that can present different crystalline lattices, the most common being the cubic, tetragonal, and orthorhombic structures. In the ideal cubic structure, 12-fold coordinated A cations are arranged on the corners of a cube surrounding a BO_6_ octahedron, where B cation sits in the body-centered positions and oxygen anions are placed in the face-centered positions [14]. Thanks to its structure, it can accommodate a large variety of metallic ions [15] that can be exploited for enhancing and tuning the dielectric and piezoelectric properties of the final material. Dopants can occupy both the A or B site depending on their charge and ionic radius [15,16]. The BT piezoelectric behavior depends on its crystalline structure. In fact, the presence of a net dipole moment is responsible for a charge displacement into the crystal lattice [17]. The cubic structure is centrosymmetric and does not present the net dipole moment. On the contrary, the tetragonal structure is elongated along the c-axis and this deformation is responsible for a local charge displacement and, consequently, for a net dipole moment [18]. The tetragonality is evaluated through the c/a ratio; the higher the tetragonality, the more pronounced the piezoelectric effect.

In the preparation of high-performance piezo-composites, one of the main issues is related to a generally poor compatibility between the ceramic nanoparticles (NPs) and the polymeric matrix, which does not allow a good dispersion of the filler, leading to a lower improvement of the piezoelectric performances. However, the surface functionalization of ceramic powders can improve the filler-matrix compatibility and enhance the dielectric and piezoelectric performance of the final product [19,20,21,22]. The yield of chemical modification depends on both the surface area and the surface reactivity of fillers, which can be improved through wet chemical syntheses of ceramic NPs.

The preparation of BaTiO_3_ nanoparticles (NPs) in a solution presents several advantages over the classic solid-state production route, in particular in terms of energy cost. The solid-state process involves the mixing of barium and titanium oxides powders, a ball milling step, and finally, calcination at very high temperatures (1100–1400 °C) [23,24]. Powders from solid state route are characterized by low homogeneity, impurities, and broad particle size distribution. Conversely, high purity homogeneous particles, with narrow size distribution and improved surface area, are obtained at lower temperatures by the sol-gel process. Accordingly, the low temperature hydrothermal method has been widely explored for the production of titanates and other perovskitic structures [25,26,27]. The sol-gel synthesis of barium titanate at both ambient pressure and hydrothermal conditions exploits different precursors and solvents. The most widely used precursors are TiO_2_ particles and Ba(OH)_2_ [28,29], titanium and barium chlorides [30], and titanium alkoxides [31,32,33]. The alkoxide-based sol-gel production route of barium titanate particles is based on the controlled hydrolysis-condensation of the precursor through the addition of organic additives, such as carboxylic acids. The process is usually carried out in a strong alkaline environment, since barium titanate formation is favored at pH > 12–13. The mechanism of formation of barium titanate starting from alkoxide precursor is still point of debate, but it is generally accepted that the addition of a carboxylic acid to an alcoholic solution of Ti(O^i^Pr)_4_ promotes the formation of a titanyl-acylate complex Ti(OR)_x_(OAc)_y_ [34,35], which reacts after the addition of the aqueous Ba-sol, leading to the formation of Ti(OH)_x_(OAc)_y_ species. The addition of a strong base (KOH, NaOH) to the Ba-Ti sol transforms Ti(OH)_x_(OAc)_y_ complexes in Ti(OH)_6_^2^− species that link to Ba^2+^ ions leads to crystalline BaTiO_3_ precipitation [28,32]. Moreover, the hydrothermal treatment can be applied to the sol−gel synthesis. This process offers several advantages over the classic sol−gel route at ambient pressure, reducing the processing time and costs and producing very fine powders with good crystallinity, homogeneity, and controlled size.

In this work, undoped and doped barium titanate nanoparticles were prepared by the sol-gel route at ambient and hydrothermal conditions. The main goal of this work is to evaluate the effect of the synthesis parameters, namely the duration of the hydrothermal treatment and the addition of dopants, on BT NPs microstructural features. Chemical, structural, and microstructural properties of the obtained powders were studied by inductive coupled plasma-optical emission spectroscopy (ICP-OES), Fourier transform infrared spectroscopy (FTIR), scanning electron microscopy (SEM), X-ray diffraction (XRD), Raman spectroscopy, and thermogravimetric and differential thermal analyses (TGA-DTA).

## 2. Materials and Methods

### 2.1. Materials

Reagent grade titanium (IV) isopropoxide Ti(O^i^Pr)_4_ (Sigma-Aldrich, Saint Louis, MO, USA, CAS 546-68-9), barium acetate Ba(CH_3_COO)_2_ (Analyticals Carlo Erba, Milan, Italy CAS 543-80-6), sodium acetate NaCH_3_COO (Analyticals Carlo Erba, Milan, Italy, CAS 127-09-3), calcium nitrate tetrahydrate Ca(NO_3_)_2_4H_2_O (Sigma Aldrich, Saint Louis, MO, USA, CAS 13477-34-4), bismuth nitrate pentahydrate Bi(NO_3_)_3_5H_2_O (Sigma Aldrich, Saint Louis, MO, USA, CAS 10035-06-0), potassium hydroxide KOH (VWR International, Milan, Italy CAS 1310-58-3), glacial acetic acid CH_3_COOH (ITW Reagents, Darmstadt, Germany, CAS 64-19-7), and absolute ethanol C_2_H_5_OH (Merck KGaA, Darmstadt, Germany, CAS 64-17-5) were employed for powders synthesis. As a comparison, commercial barium titanate powders were purchased from Acros Organics (Geel, Belgium) and the sample was labeled BT_comm.

### 2.2. Synthesis of BT Powders

BT powders were produced modifying the procedure of Hwang et al. [34,35]. The reaction was carried out under nitrogen to avoid BaCO_3_ precipitation. Ti(O^i^Pr)_4_ (0.01 moles) was reacted with glacial acetic acid and absolute ethanol in 1:4:8 molar ratio and the solution was kept under stirring for 1 h. Ba(CH_3_COO)_2_ (0.011 moles, Ba/Ti = 1.1 molar ratio) was reacted with CH_3_COOH (0.022 mol, Ba:acetic acid = 1:2 molar ratio) in 5 mL of distilled water; the clear solution was slowly added to the Ti-sol and the mixture was kept under stirring at room temperature for 1 h. Then, KOH (3N) solution was added until a pH higher than 13 was reached. The milky suspension was vigorously stirred for 7 h at 45 °C under N_2_ flux, and then was stirred at RT overnight. The powders were collected through centrifugation at 4000 rpm, washed with distilled water until neutral pH, and finally dried in an oven at 80 °C for 4–6 h and then overnight at room temperature (RT). The dried powders were annealed at 900 °C for 4 h. As-prepared and thermally treated sol-gel samples are labeled BT and BT_900, respectively.

A similar procedure was adopted for the preparation of BT powders under hydrothermal conditions. Ti and Ba sols were mixed, and the resultant solution was kept under stirring for 1 h in nitrogen. Then, KOH solution was added to the Ti-Ba solution; the milky mixture was immediately transferred into a (poly-tetrafluoroethylene) PTFE-lined stainless-steel reactor and heated at 200 °C for various times (2, 4, 6 and 8 h). The heating was stopped, and the autoclave was left to naturally cool to room temperature. The powders were collected by centrifugation, washed, and dried at 80 °C for 4–6 h and overnight at RT. As-prepared and annealed powders are labeled BT_H and BT_H_900, respectively. The doped powders were also prepared by 2 h hydrothermal treatment, by dissolving into the Ba-sol the stoichiometric amounts of sodium acetate, calcium nitrate and bismuth nitrate in order to obtain a theoretical composition Ba_0.9_X_0.1_TiO_3_ (where X = Na^+^, Ca^2+^, Bi^3+^). Undoped and doped dried powders were annealed at 900 °C for 4 h. Doped samples are labeled BT_H_X_900 (X = Na, Ca, Bi or NaBi). Table 1 summarizes sample labeling, processing conditions, eventual doping, and annealing.

### 2.3. Thermal Treatment

As-prepared powders from both sol-gel synthesis at ambient pressure and in hydrothermal conditions have been annealed at 900 °C for 4 h (heating rate 10 °C/min) in a tubular SiO_2_ furnace (Heraeus GmbH, Hanau, Germany) in order to remove any organic residual and promote the cubic to tetragonal (c⟶t) transformation.

### 2.4. Characterization

TGA-DTA curves were collected with a Netzsch STA 409 thermobalance (Netzsch-Gerätebau GmbH, Selb, Germany), between 20 and 1000 °C, at a 10 °C/min heating rate in flowing air. Chemical analysis was carried out by ICP-OES using a Spectro Ciros Instrument (Spectro Analytical Instruments GmbH & Co, Klevee, Germany). Samples (25 mg) were dissolved in 5 mL of concentrated HCl with the addition of few droplets of H_2_O_2_, and de-ionized (DI) water was added up to a final volume of 50 mL. M4M-standards were used for the quantitative analysis of Ba, Ti, and dopant elements. FTIR spectra were collected in transmission mode on KBr pellets of both as-prepared and annealed particles using a Thermo Nicolet Avatar 330 FT-IR spectrophotometer (Thermo Electron Corporation, Waltham, MA, USA) using the following parameters: range 4000–400 cm^−1^, 64 scans, resolution 4 cm^−1^. Nitrogen adsorption isotherms were acquired with a Micromeritics ASAP 2010 instrument (Micromeritics). Brunauer-Emmett-Teller (BET) and Barrett-Joyner-Halenda (BJH) models were used, respectively, for the determination of specific surface area and pore size distribution. SEM micrographs of annealed powders were acquired with a Carl Zeiss Gemini Supra 40 field emission scanning electron microscope (FE-SEM) (Zeiss, Oberkochen, Germany), with an accelerating voltage of 10 kV at 20,000×, 50,000×, and 80,000× magnification using secondary electrons as main signal. Particle size analysis was performed by ImageJ software (https://imagej.nih.gov/ij/, accessed on 29 July 2021). XRD analyses were performed with a Rigaku III-D Max diffractometer (Rigaku, Tokyo, Japan), using CuKα radiation and a curved graphite monochromator in the diffracted beam. All samples were analyzed using the following parameters: 40 kV, 30 mA, 10° < 2θ < 90°, step 0.05°, acquisition time 2 s. The phase analysis was performed using JADE8 software (Materialsdata Inc., MDI, Livermore, CA, USA). Patterns were then processed with MAUD software [36] (https://maud.radiographema.eu, accessed on 18 March 2021) to evaluate the phase composition, lattice parameters, and crystallite size. In order to take into proper account the instrumental broadening of the peaks during the deconvolution procedure, a calibration was performed using KCl as a reference material. Raman measurements were carried out using a LabRAM Aramis (Horiba Jobin-Yvon) equipped with an optical microscope and a 100× objective. A diode-pumped solid-state laser source of 532 nm was used for the excitation of the Raman signal that was detected with an air-cooled charge coupled device. A diffraction grating with 1800 lines mm^−1^ was used for the collection of all Raman spectra with an overall spectral resolution of 1 cm^−1^. Raman spectra have been acquired with an overall acquisition time of 4 s by setting the laser power at 3 mW.

## 3. Results and Discussion

**Figure 1:**
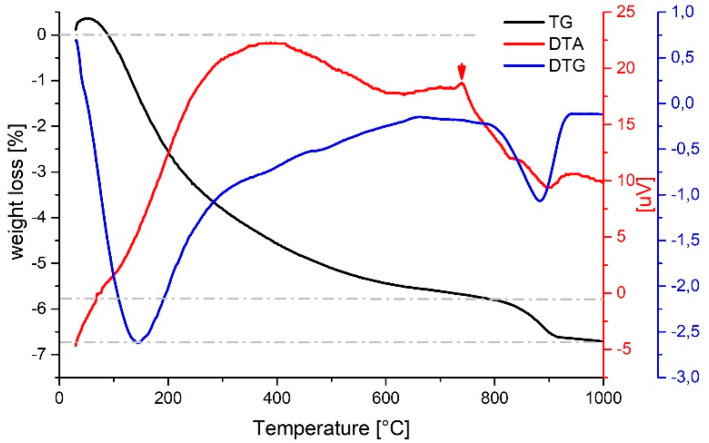
TGA−DTA curves of BT powders.

Preliminary experiments have been done in order to optimize the sol−gel synthesis parameters, in particular the molar ratio between Ti and Ba precursors (Ba/Ti ratio). It was found that that Ba/Ti ratio equal to 1 led to the formation of several metastable (low barium) Ba-Ti oxides, such as BaTi_2_O_5_, Ba_2_Ti_5_O_11_, and Ba_4_Ti_13_O_30_, in agreement with previous reports [37,38]. On the contrary, a Ba/Ti ratio slightly higher than 1 reduced the formation of these undesired phases leading to pure BaTiO_3_. Accordingly, in this work, a Ba/Ti ratio equal to 1.1 was used for sol−gel syntheses both at ambient pressure and in hydrothermal conditions. For doped samples, the ratio (Ba + dopant)/Ti = 1.1 was used, since the dopant element partially substitutes the Ba-cation in the perovskite A-site. As-prepared powders were annealed in order to promote the formation of tetragonal phase. Figure 1 shows the results of TGA−DTA analysis of BT powders in the range 20–1000 °C. Two weight loss steps are visible in the TG curve: the main one (about 6%) is found in the range 20–800 °C, and it is attributed to both release of organic residuals and condensation of surface hydroxyl groups; between 800 and 900 °C, a second weak effect (about 1%) is probably ascribable to the release of lattice hydroxyl defects typically present in samples prepared by wet chemical methods [39]. The sample appears thermally stable above 900 °C. According to Ashiri [40], who studied the thermal evolution of sol-gel barium titanate powders, above 800 °C, barium carbonate is removed, and the BT phase transformation is completed. The DTA curve shows an exothermic peak centered at 386 °C, which is attributed to the combustion of organics and the decomposition of BaCO_3_ and intermediate phases [41]. An exothermal effect is observed at 740 °C and attributed to the crystallization of BT tetragonal phase [42]. In fact, the cubic-to-tetragonal phase transformation is reported at around 850 °C [40], but the tetragonal phase starts to appear above 600 °C [43] probably due to intermediate structures in a local environment of the lattice. According to the result of thermal analyses, annealing at 900 °C, with isothermal steps of 4 h, was selected for all samples in order to decompose unwanted phases and complete the cubic-to-tetragonal transformation.

**Figure 2:**
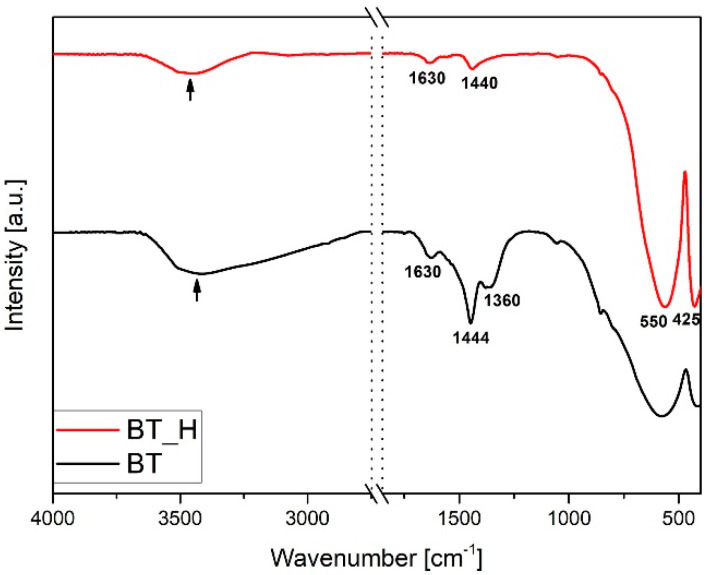
FTIR spectra of powders produced by sol−gel method at ambient pressure (BT) and hydrothermal conditions (BT_H).

Figure 2 shows the FTIR spectra of as-prepared BT powders produced via sol−gel synthesis both at ambient pressure and under hydrothermal conditions. Typical M-O vibrations are found below 800 cm^−1^. In particular, broad signals due to Ti-O stretching and Ti-O-Ti bending signals are found, respectively, at 550 and 425 cm^−1^ [40,44] in both samples, but they are narrower and more intense in the BT_H spectrum.

**Figure 3:**
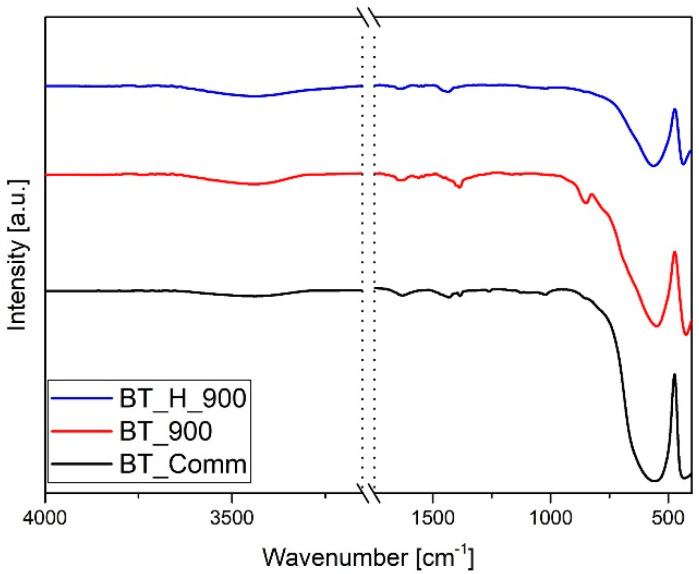
FTIR spectra of BT_comm, BT_900, and BT_H_900.

In the BT spectrum a complex band is present in the range 1700–1250 cm^−1^. The main component at 1444 cm^−1^ [45], due to the normal vibration of carbonate groups, is overlapped to the signals of residual acetates in the range 1550–1300 cm^−1^ [46,47,48]. The band at 1630 cm^−1^ is due to the water scissoring vibration of adsorbed water. In the corresponding OH stretching band centered at 3500 cm^−1^, the high wavenumber component is attributable to the BT hydroxyl groups. The intensity of the carbonate signal (1440 cm^−1^) is strongly reduced in the BT_H spectrum and no evident signals of residual acetates can be appreciated. The FTIR spectra of powders annealed at 900 °C are reported in Figure 3. The thermal treatment leads to the disappearance of residual organic groups and strong reduction of carbonates in agreement with TGA−DTA results [40,49]. It is worth noting that carbonate vibrations also appear in the spectrum of the commercial product, reported in Figure 3 for the sake of comparison. The characteristic titanate signals are narrower in both sol-gel samples with respect to the commercial product. The spectra of annealed nanoparticles are also representative of the doped samples.

### 3.1. Effect of Hydrothermal Reaction Time

**Figure 4:**
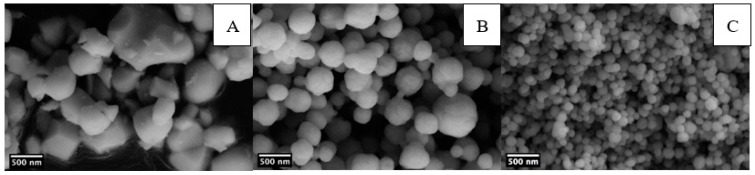
SEM micrographs of BT_comm (**A**), BT_900 (**B**), and BT_H_900 (**C**).

SEM images on barium titanate powders were obtained to evaluate the size, the distribution, and the morphology of particles. Figure 4 shows the SEM micrographs of commercial particles and sol-gel powders, prepared both at ambient pressure and in hydrothermal conditions.

SEM images show that fine powders from synthesis at ambient pressure form through the agglomeration of primary particles [28,35], which is also valid for hydrothermal samples. Table 2 summarizes the results of the particle analysis performed by ImageJ software. Particles obtained by sol-gel synthesis at ambient conditions (BT_900 sample, mean size 376 nm) are spherical smaller in size and with narrower distribution with respect to commercial BT particles (BT_comm sample, mean size 496 nm), which display less regular morphology. The 2 h hydrothermal treatment (BT_H_900 sample, mean size 117 nm) leads to a relevant decrease in particle dimensions, and even narrower size distribution was obtained as compared with BT_900 sample.

**Figure 5:**
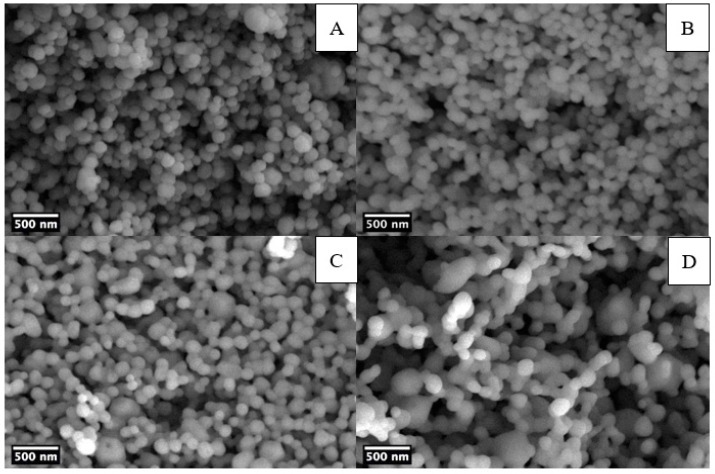
SEM micrographs of BT particles in hydrothermal conditions at different processing times: 2 h (**A**), 4 h (**B**), 6 h (**C**), and 8 h (**D**).

The effect of the duration of the hydrothermal processing was studied acquiring the SEM micrographs of BT nanoparticles produced with 2 h, 4 h, 6 h, and 8 h reaction time (Figure 5). Increasing the hydrothermal reaction time from 2 h to 8 h does not have remarkable effects on the particle size [50]. The mean particle size was found only to slightly increase from 117 to 162 nm with a corresponding broadening of the size distribution (Table 2). Up to 6 h reaction time NPs are spherical, whereas the sample obtained after 8 h hydrothermal treatment presents bigger and more elongated particles, with the appearance of necks. From these results, it appears that a 2 h hydrothermal treatment is preferable, as it saves time and produces spherical particles with a narrow size distribution compared to higher reaction times.

**Figure 6:**
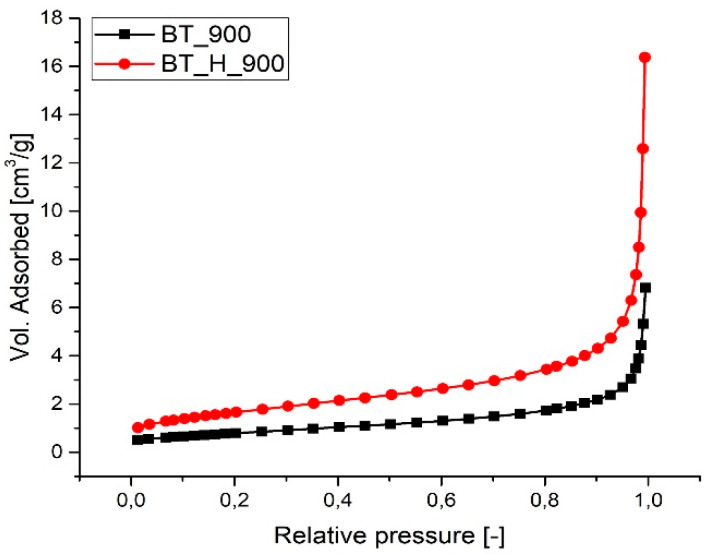
N_2_ adsorption isotherms of BT_900 and BT_H_900.

Figure 6 shows the N_2_ physisorption isotherms of barium titanate produced at both ambient pressure and in hydrothermal conditions (2 h) and annealed at 900 °C. Powders display type II isotherms [51]. The BET-specific surface area (SSA) values of BT_900 and BT_H_900 samples are, respectively, 2.87 m^2^/g and 5.95 m^2^/g, which are consistent with the main contribution of the geometrical surface in dense particles. However, the BJH model applied to the adsorption curve indicates the presence of some porosity. This porosity has a bimodal behavior with pore diameters ranging from 2 to 10 nm and from 13 to 90 nm.

Assuming spherical particles, the mean particle size was calculated using the following formula:

D = 6000/(SSA_BET_ × ρ) (1)

where D is the mean diameter (in nm), SSA is the value of the BET specific surface area (in m^2^/g) and ρ is the density of barium titanate (theoretical, 6.02 g/cm^3^). Mean diameter was calculated to be equal to 347 nm for BT_900 and 167 for BT_H_900. These dimensions, within the experimental error, are comparable with those measured from SEM micrographs.

**Figure 7:**
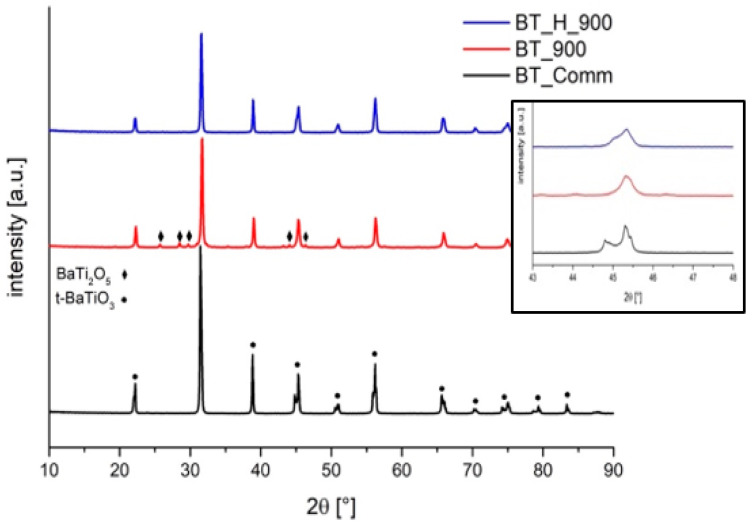
XRD patterns of BT_comm, BT_900, and BT_H_900. The signal at 2θ = 45° is shown in the figure inset.

X-ray diffraction patterns (Figure 7) were acquired on both annealed particles and on commercial barium titanate in order to evaluate phase composition, lattice parameters, crystallinity, and crystallite size.

Barium titanate tetragonal phase (PDF 5-626) is evidenced by the presence of a doublet at 2θ = 45° [52,53], whereas the cubic phase (PDF 31-174) is recognized by a single peak at 2θ = 45°and the coexistence of the two phases by a main peak combined with a shoulder. As shown in Figure 7, XRD pattern of commercial powders clearly presents a doublet, while annealed powders from sol−gel both at ambient pressure and hydrothermal condition present an intermediate situation, with the main peak and a shoulder (Figure 7, inset), evidence of the coexistence of both tetragonal and cubic phase. No additional phases are found in hydrothermal powders over cubic and tetragonal. On the contrary, BT_900 sample presents traces of metastable BaTi_2_O_5_ oxide (monoclinic PDF 8-368). As explained by Ritter et al. [37], several oxides, such as BaTi_2_O_5_, BaTi_5_O_11_, and Ba_2_TiO_4_, can form, depending on the Ba/Ti ratio, which crystallize at 700 °C and are quite stable up to 900–1100 °C. The mechanisms behind the formation of these oxides are very complex but it is supposed that both water and acetic acid amount used in the sol synthesis play a role. On the other hand, BT powders produced in hydrothermal conditions are made only by BaTiO_3_, and tetragonal and cubic phase coexist in all samples produced. In order to evaluate the relative amounts of tetragonal and cubic phase and the lattice parameters, Rietveld analysis was applied to XRD patterns [36,54]. Table 3 shows the calculated relative weight amounts of tetragonal and cubic phase in the undoped annealed samples.

**Figure 8:**
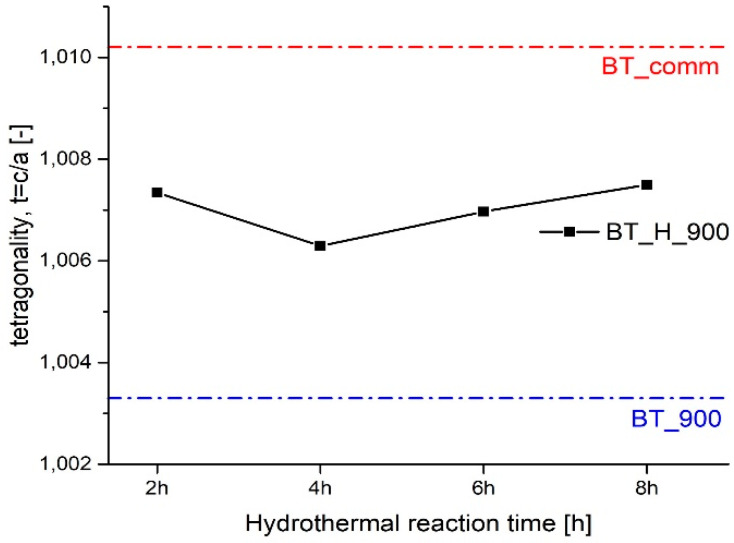
Trend of tetragonality as a function of the hydrothermal reaction time.

The relative amount of tetragonal phase is about 75% regardless of the hydrothermal reaction time and this evidence confirms that it is worth to use the shorter hydrothermal treatment (2 h), considering that it leads also to better results in terms of particles morphology according to SEM observations. Table 3 also presents crystallite size, lattice parameters, and tetragonality calculated for the undoped samples. The commercial powders present crystallites of 114 nm, whereas crystallites of 46 nm are found for BT_900. The evaluation of the crystallite dimensions of hydrothermal powders is affected by the coexistence of cubic and tetragonal lattices. The fitting of experimental data was performed using the reference powder diffraction files of both tetragonal and cubic crystalline phases. Unfortunately, the overlapping of the characteristic diffraction peaks of the tetragonal and cubic crystalline makes it more difficult to assess the crystallite size for the cubic structure, which is the minority phase, as demonstrated by the higher standard deviation values reported in Table 3. However, the residual weight percentage (Rwp) is sufficiently low to consider reliable the results, which suggests a constant trend in size for the tetragonal phase and an increase for the cubic with the increase of the duration of the hydrothermal treatment. To evaluate the dependence of the results on the structural models, the refinement was also performed using separately the PDF of the tetragonal and cubic structures. The results confirmed for the tetragonal phase crystallite sizes in the range 70–75 nm; smaller crystallites (42–52 nm) were calculated for the cubic phase, but these latter results are accompanied by a higher Rwp [%], strengthening the assumption that the coexistence of phases is the cause of the high error in the assessment of crystallite size of the minor phase. The key parameter to have high piezoelectric performance are: tetragonality, amount of tetragonal phase, grain size, domain size, and orientation [55,56]. In accordance with the literature, the highest achievable value of tetragonality t is 1.010 [57] through the solid-state route. Table 3 shows the lattice parameters of all samples. Powders from hydrothermal synthesis display much higher values of tetragonality (1.006–1.007) with respect to ambient pressure sample (1.003); their value is not so far from the maximum, even if treated at relatively low temperatures (900 °C with respect to 1100–1400 °C typical of solid-state reactions). The hydrothermal reaction time seems to have little effect on lattice parameters and, consequently, on tetragonality, as shown in Figure 8.

**Figure 9:**
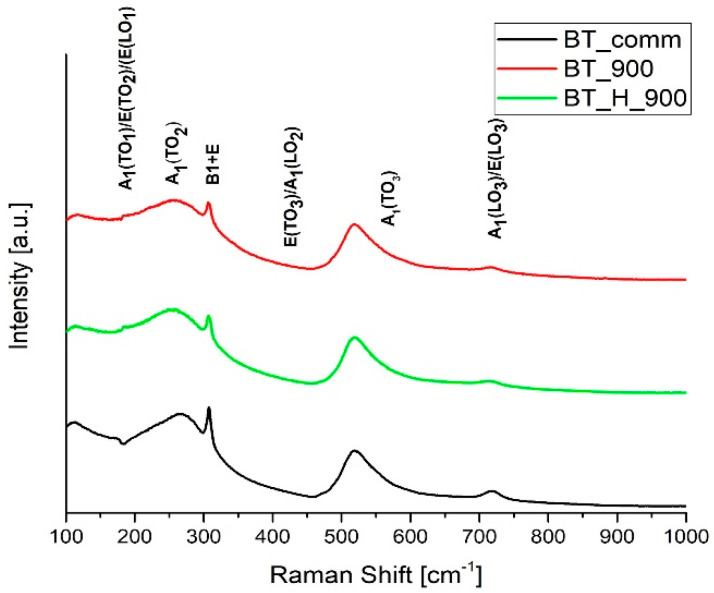
Raman spectra of BaTiO_3_ samples: BT_comm (black line), BT_900 (red line), and BT_H_900 (green line).

Raman spectroscopy is a highly sensitive spectroscopic technique to probe the local structure of atoms in materials; hence, Raman studies have been carried out to investigate the structure as a function of the conditions employed to synthetize the BaTiO_3_ nanoparticles. Figure 9 shows Raman spectra acquired on the samples labeled BT_comm, BT_900, and BT_H_900. Analyzing the spectra reported in Figure 9, it is possible to distinguish the typical Raman-active modes for tetragonal BaTiO_3_: 4E(TO + LO) + 3A_1_(TO + LO) + B_1_(TO + LO). The phonon modes corresponding to Raman bands are assigned as proposed by Dixit et al. [58]. Moreover, the observed anti-resonance effect at 183 cm^−1^ as an interference feature, is attributed by Scalabrin et al. to a coupling between the sharp A_1_(TO_1_) and broad A_1_(TO_2_) modes [59]. Furthermore, it is well known that the BaTiO_3_ tetragonal phase presents Raman scattering bands at around 250, 520, and 720 cm^−1^ and a sharp peak at around 306 cm^−1^ [60].

According to these assignments, the Raman spectra confirm that the BT samples have a tetragonal structure, in agreement with XRD results. Furthermore, according to the intensity of the signal at 306 cm^−1^, higher tetragonality of commercial powders is confirmed, as also shown from the XRD analysis.

### 3.2. Effect of Doping

The effect of doping on the powder microstructural features was also analyzed. Table 4 summarizes the calculated molar percentages of Ba and dopant cations for the undoped and the doped samples and reports the Ba/Ti molar ratio, obtained from ICP−OES measurements. According to the elemental analysis, both undoped and doped samples are barium deficient even if a Ba/Ti molar ratio 1.1 was used in the synthesis. This is in accordance with the literature, which points out the difficulty of obtaining stoichiometric BaTiO_3_ powders through chemical syntheses performed in strongly basic conditions [61,62,63,64]. Elemental analysis (Table 4) confirmed the presence of Ca, Na, and Bi dopants in the samples, but their quantity is lower with respect to the nominal one, meaning that the doping effectiveness is lower than expected. In addition, K is detected in all samples as a consequence of the use of KOH in the synthesis. K traces are found when doping with a divalent ion or a combination of monovalent and trivalent ions; on the contrary, high K content is found in Na- and Bi-doped samples. Moreover, sodium is also detected in undoped and Ca- and Bi-doped samples, due to its presence in KOH starting solution. According to the literature, K^+^, Na^+^, Ca^2+^, and Bi^3+^ ions substitute Ba^2+^ in the A-site of the perovskitic structure, mainly because of the ionic radius dimension [65,66,67,68,69,70,71]. According to the values reported in Table 4, the Ba/Ti ratio decreases in the doped samples with respect to undoped BT. This is a direct consequence of the Ba substitution by the dopant cations. In details, Ca^2+^ effectively substitute Ba^2+^, due to the maintenance of the charge balance, thus leading to the lowest Ba/Ti ratio. In the case of the Na-doped sample, the amount of Na is limited but a large doping contribution comes from K probably due to the ionic radius that is closer to that of Ba. In order to keep the charge balance, the substitution of divalent cations with monovalent cations in A-site leads to a significant decrease of Ba/Ti ratio. The case of the Bi-doped sample deserves two comments. Taking into consideration the charge balance, the amount of Bi^3+^ should be necessary limited, but this sample also displays a large amount of potassium, thus justifying the contained variation in Ba/Ti ratio. Similar considerations can be made for BT_H_NaBi_900 sample. Both charge balance and dopants size could affect the different amount of K detected in the samples. As reported above, the charge balance should play a crucial role but dopants size could also affect the extent of substitution, since this has a relevant effect on unit cell deformation. However, the K amount in the Bi-doped sample is surprisingly high and will be a matter of future investigation.

**Figure 10:**
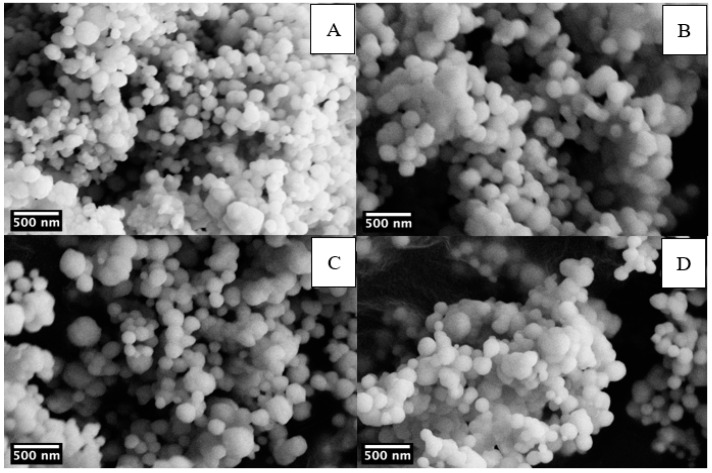
SEM micrographs of BT doped particles: BT_H_Na_900 (**A**), BT_H_Ca_900 (**B**), BT_H_Bi_900 (**C**), and BT_H_NaBi_900 (**D**).

Figure 10 shows the SEM micrographs of the doped samples. It can be seen that particles are spherical and quite homogeneous. Table 5 summarizes the results of particle size analysis performed with the software ImageJ. Particles have a similar dimension, ranging from 134 to 149 nm, regardless of the nature of doping. However, if compared with the BT_H_900, the doped samples present a slightly bigger mean size and broader distribution (higher standard deviation). 

**Figure 11:**
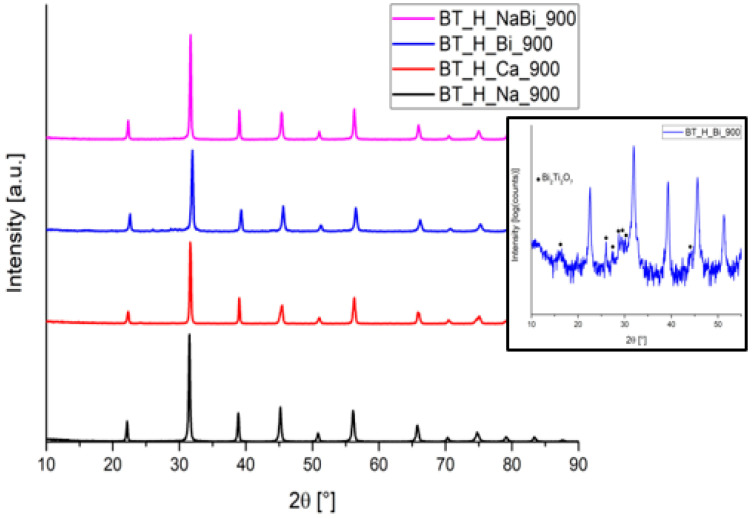
XRD patterns of doped BaTiO_3_ samples: BT_H_Na_900 (black line), BT_H_Ca_900 (red line), BT_H_Bi_900 (blue line), and BT_H_NaBi_900 (pink line). Inset: BT_H_Bi_900 pattern shown in log scale to highlight the presence of secondary phase traces.

XRD patterns of the doped samples confirm the presence of the BaTiO_3_ phase in all samples, as shown in Figure 11. Generally, no additional phases are found. Only in the case of the Bi-doped sample, as also reported by Zhou et al. [68], traces of Bi_2_Ti_2_O_4_ (PDF 32-0118) are detected (<0.5 wt.% from Rietveld analysis). Table 6 summarizes the results of the XRD pattern analysis of the doped BT powders. It is worth to note that all the simulations made by MAUD software present a reliable goodness of fitting and Rwp (%). The Ca addition does not affect both relative amount of tetragonal phase and tetragonality, which appear almost unchanged with respect to the values calculated for BT_H_900 sample. The substitution of Ba^2+^ with a smaller divalent cation leads to a decrease of both unit cell parameters, therefore keeping the t value (1.0070) almost unchanged. According to the literature [70], the presence of heterovalent ions leads to a decrease of the tetragonality parameter, as observable in Table 6. In case of Na-doping, the quantitative analysis evidenced that lower amount of tetragonal phase was formed after annealing. On the other hand, Bi-doping and the combined NaBi-doping have an opposite effect: when both bismuth and sodium are added, the relative amount of tetragonal phase is higher than when bismuth is standalone. For both samples, a decrease in tetragonality values is observed due to a more pronounced shortening of c length. Finally, concerning the crystallite size dimension, it can be observed that dopant addition leads to variation depending on the nature of the element. Calcium doping produces an increase in size both for tetragonal and cubic phases, whereas Bi and Bi/Na addition leads to decreasing the crystallite dimension. Additionally, here, the standard deviation associated to the cubic phase is higher with respect to the one associated with the main tetragonal phase.

**Figure 12:**
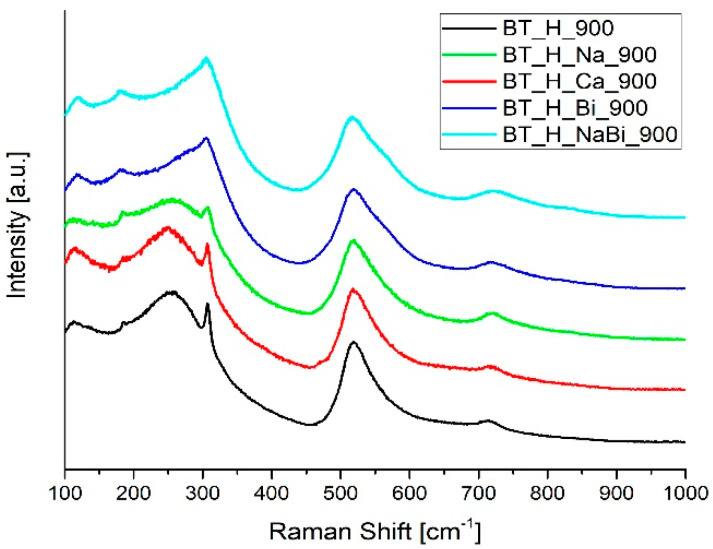
Raman spectra of cation−doped BaTiO_3_ systems: (black line) undoped sample, (red line) BT_H_Ca_900 sample, (green line) BT_H_Na_900, (blue line) BT_H_Bi_900, (light blue line) BT_H_NaBi_900.

A complementary study, using the Raman Spectroscopy technique, has been carried out to probe the effect of doping (Na^+^, Ca^2+^ and Bi^3+^) on the BaTiO_3_ structure. Figure 12 shows the Raman spectra of cation-doped BaTiO_3_: (a) in sample BT_H_Ca_900, it is not possible to appreciate any significant variation between reference and the Ca^2+^ doped sample, suggesting that in this case, the Ca addition does not modify the structural features of the nanoparticles, in agreement with what was established by the XRD analysis, (b) in the sample with Na^+^ doping (green line), a decrease of the intensity peak is evident at 306 cm^−1^ related to the B+E mode, indicating a lower amount of tetragonal phase with respect to the blank one, as evidenced by Hayashi et al. [60] and confirmed by XRD quantitative analysis; (c) finally, for both BT_H_Bi_900 and BT_H_NaBi_900 samples, the Bi^3+^ doping produces a modification in the structure with a shift towards higher wavenumbers of the modes A_1_(TO) at 250–270 cm^−1^, and A_1_, E(TO) at 510–520 cm^−1^ with respect to the undoped BaTiO_3_, in agreement to what was already reported by Strathdee et al. [72]. 

## 4. Conclusions

In conclusion, BaTiO_3_ nanoparticles were produced both by traditional sol-gel synthesis and under hydrothermal conditions. The hydrothermal treatment presented some evident advantages over the sol−gel route at ambient pressure. Nanoparticles from hydrothermal synthesis were spherical, more homogeneous, and with narrower size distribution with respect to classic solid−state route or traditional sol-gel synthesis. Furthermore, it was demonstrated that a longer duration of the hydrothermal process causes a broadening in particle size distribution combined with the appearance of some necks among particles. FTIR showed the presence of organic residuals in powders prepared by sol-gel synthesis at ambient pressure, which were removed after annealing at 900 °C. XRD analysis indicated a high amount of tetragonal phase and high values of tetragonality, even if they were annealed at a relatively low temperature (900 °C) with respect to the conventional production method. These findings were also confirmed by Raman spectroscopy. The hydrothermal reaction time (2–8 h) was demonstrated to have almost no effect on the amount of tetragonal phase and on tetragonality. Considering also the morphological analysis performed through SEM observation, these results led to the conclusion that a 2 h hydrothermal process is time-saving and produces spherical particles with narrower size distribution and high value of tetragonality compared to higher reaction time.

Finally, NPs doping was performed with Na, Ca, and Bi and the effect of dopant on microstructure and cubic-to-tetragonal phase transformation was evaluated. In particular, the addition of both Na and Bi ions improved the transformation and led to decreasing crystallite dimensions, while Ca-doping did not result in appreciable variations of BT powder properties. Tetragonality was found to decrease in all doped samples, in agreement with the literature, even if Ca doping did not alter this value significantly. The effect of dopants on the microstructural properties of barium titanate depends both on charge and ionic radius of doping ions. The balance of unit cell deformation and charge imbalance seems to rule the dopant concentration. Due to the sample preparation procedure, potassium was detected in all samples in different amounts, depending on the nature of the dopant element. Furthermore, the particle dimension for doped samples was found to be slightly higher with respect to the undoped sample (BT_H_900).

## Figures and Tables

**Table 1:** Sample labeling and description of production method.

Sample	Hydrothermal Treatment	Doping *	Annealing
BT	-	-	-
BT_900	-	-	900 °C, 4 h
BT_H	200 °C, 2 h	-	-
BT_H_900	200 °C, 2 h	-	900 °C, 4 h
BT_H4h_900	200 °C, 4 h	-	900 °C, 4 h
BT_H6h_900	200 °C, 6 h	-	900 °C, 4 h
BT_H8h_900	200 °C, 8 h	-	900 °C, 4 h
BT_H_Na_900	200 °C, 2 h	Na (10% mol.)	900 °C, 4 h
BT_H_Ca_900	200 °C, 2 h	Ca (10% mol.)	900 °C, 4 h
BT_H_Bi_900	200 °C, 2 h	Bi (10% mol.)	900 °C, 4 h
BT_H_NaBi_900	200 °C, 2 h	Na (5% mol.); Bi (5% mol.)	900 °C, 4 h

* Doping % is expressed with respect to the total amount of element A in ABO_3_ structure.

**Table 2:** Results of the particle size analysis from SEM micrographs.

	BT_comm	BT_900	BT_H_900	BT_H4h_900	BT_H6h_900	BT_H8h_900
Mean (nm)	496 ± 178	376 ± 99	117 ± 27	127 ± 31	131 ± 40	162 ± 56

**Table 3:** Quantitative phase analysis, crystallite size, and lattice parameters of undoped BT powders.

Sample	BT_comm	BT_900	BT_H_900	BT_H4h_900	BT_H6h_900	BT_H8h_900
Phase [wt.%]	Tetra	Tetra–Other	Tetra–Cubic	Tetra–Cubic	Tetra–Cubic	Tetra–Cubic
	100	85(2)–15(2)	75(1)–25(1)	73(3)–27(3)	74(3)–26(3)	78(2)–22(2)
Crystallite (std.dev) [nm]	114(1)	46(1)–na	77(4)–82(14)	75(1)–114(18)	73(3)–92(16)	75(4)–171(46)
a [Å] (tetra)	3.9995	4.0105	4.0039	4.0053	4.0049	4.0042
c [Å] (tetra)	4.0393	4.0239	4.0333	4.0305	4.0318	4.0342
t = c/a (tetra)	1.010	1.0033	1.0074	1.0063	1.0069	1.0075
Rwp [%]	15.09	12.94	18.05	20.00	16.04	15.43

**Table 4:** Experimental molar composition of BaTiO_3_-doped samples from ICP-OES results.

	Ba/Ti mol Ratio	mol% Ba	mol% Ti	mol K%	mol Na%	mol Ca%	mol Bi%
BT_H_900	0.86	45.1	52.2	2.6	0.1	-	-
BT_H_Na_900	0.76	40.0	52.6	5.5	1.9	-	-
BT_H_Ca_900	0.74	38.6	52.1	0.2	1.7	7.6	-
BT_H_Bi_900	0.82	37.9	46.3	10.1	3.4	-	2.3
BT_H_NaBi_900	0.81	41.7	51.6	0.3	2.0	-	4.4

**Table 5:** Results of the particle size analysis from SEM micrographs for the doped samples.

	BT_H_Na_900	BT_H_Ca_900	BT_H_Bi_900	BT_H_NaBi_900
**Mean (nm)**	134 ± 37	149 ± 33	146 ± 39	141 ± 37

**Table 6:** Quantitative phase analysis, crystallite size, and lattice parameters of doped BT powders.

Sample	BT_H_900	BT_H_Na_900	BT_H_Ca_900	BT_H_Bi_900	BT_H_NaBi_900
Phase [wt.%]	Tetra–Cubic	Tetra–Cubic	Tetra–Cubic	Tetra–Cubic	Tetra–Cubic
	75(1)–25(1)	56(2)–44(2)	76(2)–24(2)	84(4)–16(3)	90(1)–10(1)
Crystallite (std.dev) [nm]	77(4)–82(14)	27(1)–57(1)	62(1)–112(30)	47(2)–12(1)	60(1)–7(1)
a [Å] (tetra)	4.0039	4.0101	4.0007	4.0020	4.0101
c [Å] (tetra)	4.0333	4.0331	4.0286	4.0155	4.0221
t = c/a (tetra)	1.0074	1.0057	1.0070	1.0034	1.0030
Rwp [%]	18.05	17.00	21.49	14.89	17.89

## Data Availability

The data presented in this study are available on request from the corresponding author.
